# A TaqMan-based real-time PCR assay for specific detection of novel duck reovirus in China

**DOI:** 10.1186/s12917-020-02523-z

**Published:** 2020-08-25

**Authors:** Shuai Zhang, Weihua Li, Xiaodong Liu, Xudong Li, Bin Gao, Youxiang Diao, Yi Tang

**Affiliations:** 1 College of Animal Science and Technology, Shandong Agricultural University, 61 Daizong Street, Tai’an, 271018 Shandong Province China; 2 Shandong Provincial Key Laboratory of Animal Biotechnology and Disease Control and Prevention, Tai’an, 271018 Shandong China; 3 Shandong Provincial Engineering Technology Research Center of Animal Disease Control and Prevention, Tai’an, 271018 Shandong China; 4 College of Animal medical, Qingdao Agricultural University, Qingdao, 266109 Shandong China; 5 Qingdao Yibang Bioengineering Co. Ltd., Qingdao, 266000 Shandong China

**Keywords:** Novel duck reovirus, σC gene, Real-time PCR assay, TaqMan-based probe, Detection method

## Abstract

**Background:**

In China, Newly emerging duck reovirus (NDRV) variants have been causing major disease problems in cherry valley ducks. NDRV has the potential to cause high morbidity and 5–50% mortality rates. Severe hemorrhagic-necrosis in the liver and spleen were commonly seen in NDRV affected ducks. The availability of upgraded methods for rapid diagnosis of newly emerging DRV variants is crucial for successful DRV infection control and prevention.

**Results:**

In this study, we present a TaqMan-based real-time PCR assay (RT-qPCR) for the detection of NDRV infection. Using the conserved regions within the NDRV genome, we designed the specific primers and probe. The lower limit of detection for NDRV infection was 10 copies/μL (Ct values: 38.3) after the optimization of the RT-qPCR conditions. By cross-checking with other duck viral pathogens, no cross-reactivity was observed confirming the assay was highly specific for the detection of NDRV. Reproducibility of the RT-qPCR was confirmed by intra- and inter-assay variability was less than 2.91%(Intra-assay variability of Ct values: 0.07–1.48%; Interassay variability of Ct values: 0.49–2.91%). This RT-qPCR and conventional PCR (cPCR) detected one hundred and twenty samples of NDRV infection from different regions. The result shows that the positive rates were 94.17 and 84.17% respectively. The detection rate of RT-qPCR rapid detection assay was 10% higher than that of the cPCR method.

**Conclusion:**

This research developed a highly sensitive, specific, reproducible and versatile of RT-qPCR for quantitatively detecting NDRV. It can be used to study the pathogenesis and epidemiology investigation of NDRV.

## Background

Duck reovirus (DRV), a fatal aquatic bird pathogen, is a member of the genus *Orthoreovirus* in the family *Reoviridae* [1]. Muscovy Duck Reovirus (MDRV) was first identified in South Africa [2], and then was isolated in France [3], Israel [1], Italy [4] and Germany [5]. In China, DRV was firstly noted in 1997 [6]. It showed a series of clinical symptoms, including general weakness, diarrhea, growth retardation, pericarditis, swollen liver and spleen covered with small white necrotic foci [7–9]. Based on electrophoretic mobility, the DRV contains 10 double-stranded RNA (dsRNA) genome segments which can be separated in to three size classes, including large (L1-L3), medium (M1-M3) and small (S1-S4) [10–14].

In recent years, a new duck reovirus disease was detected in China. The disease could affect different breeds of ducks and goslings. The main characteristic of the disease is hemorrhagic-necrosis in the liver and spleen [10, 12, 13, 15, 16]. The novel duck reovirus is distinct from previous MDRV isolates [9]. Thus, to distinguish it from the “classical” MDRV, the reovirus has been categorized as “novel” duck reovirus (NDRV) [15, 17]. Recently, Related research found that a new variant of a duck orthoreovirus that is significantly different from any previously reported waterfowl-derived othoreovirus, causing duck spleen necrosis [18]. The complete sequences of the 10 genome segments of NDRV have been completely determined [11]. NDRV S1 segment is similar to avian reovirus (ARV), but it is distinct from classical MDRV. NDRV S1 contains three sequential overlapping ORFs, encoding p10, p18, and σC, but in MDRV p10 and σC proteins are encoded by the S4 segment and p18 is not present [10–13, 16].

Rapid detection methods are the key for successful NDRV infection control. For many years now, quantitative real time PCR has been a standard diagnostic method, due to its rapid nature, sensitivity, reproducibility, and the reduced risk of false positives from the mispriming of the amplification primers. For viral epidemiological surveillance and pathogenesis studies, this method had been widely used [19–22]. Currently there are not any reports on a TaqMan-based real-time PCR assay for the specific detection of the novel reovirus infection. Thus, it is critical to develop the TaqMan-based real-time PCR assay of detecting NDRV infection.

In this study, we isolated novel reovirus distinct from previous duck reoviruses identified in China and developed a TaqMan probe-based RT-qPCR method which was developed for precise detection of NDRV infection based on specific primers and probe. The specific primers and probe were designed by targeting the conserved region of the NDRV S2 gene after bioinformatics analysis. The TaqMan-based real-time PCR assay was utilized extensively for virus pathogenesis studies and epidemiological investigations of NDRV.

## Results

### The selection and design of primers and probe

**Fig. 1:**
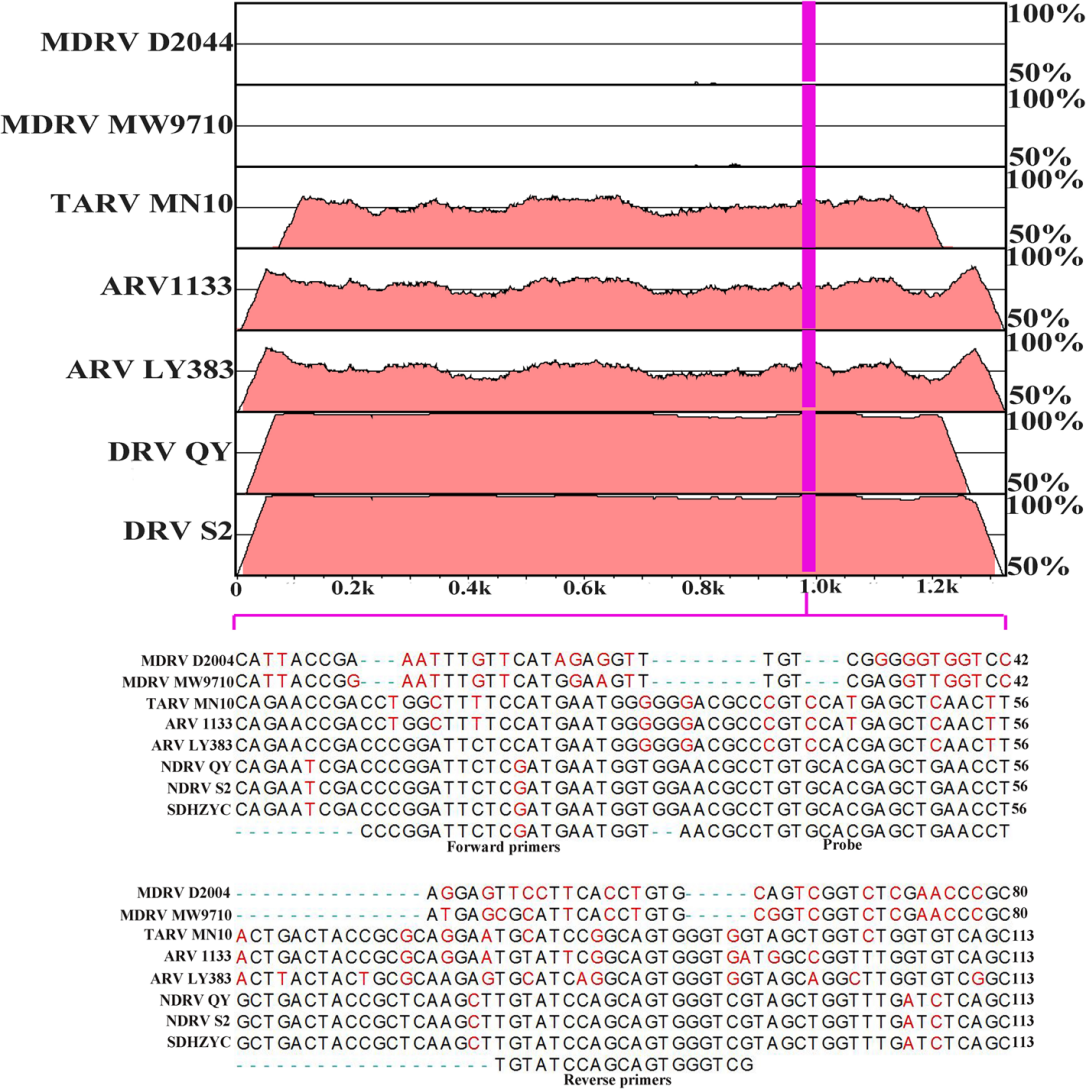
S2 genome segment alignment of different avian reovirus by mVISTA method (upper figure) and ClustalW method (lower figure); The figures illustrate alignment results of the DRV (HN5d, QY, and S2) in comparisons with ARV (MN10, 1133, and LY383) and MDRV strains (D2044 and MW9710) retrieved from GenBank; Areas in pink color represent ≥95% similarities; and areas in white represent < 95% similarities. The scale bar measures approximate length of the concatenated genome. The lower figure shows the primers and probe were shown according to the alignment result

The probe and primers used in the study were designed based on the S2 gene of NDRV (Table 1). The primers can amplify fragments of 85 bp in length. In Fig. 1, the S2 genome segment alignment of different avian orthoreoviruses were compared by mVISTA method and ClustalW method. The results showed that the primers and probes failed to align with sequences of other poultry reoviruses. Also, primers and probe were verified by the Basic Local Alignment Search Tool (BLAST, https://blast.ncbi.nlm.nih.gov/Blast.cgi) for specificity analysis [18].

**Table 1:** Primers and probe for NDRV detection used in this study

RT-PCR	Oligo	Sequence (5′-3′)	Length (bp)	Positions (Segment)
Realtime	Forward primer	CCCGGATTCTCGATGAATGGT	21	958–978(S2)
	Probe	FAM-AACGCCTGTGCACGAGCTGAAC-3′-TAMRA^a^	22	981–1022(S2)
	Reverse primer	CGACCCACTGCTGGATACAAG	21	1022–1042(S2)
σC full-length	Forward primer	ATGGATCGCAACGAGGTGATAC	22	571–592(S1)
	Reverse primer	CTAGCCCGTGGCGACGGT	18	1519–1536(S1)
σC conventional	Forward primer	TGAGACGCCTGACTACGATT	20	707–726(S1)
	Reverse primer	ATGCTTGGAGTGAGACGACT	20	1056–1075(S1)

^a^FAM, 6-carboxy-fluoresce; TAMRA, 5-Carboxytetramethylrhodamine

### Phylogenetic analysis of σC genes

**Fig. 2:**
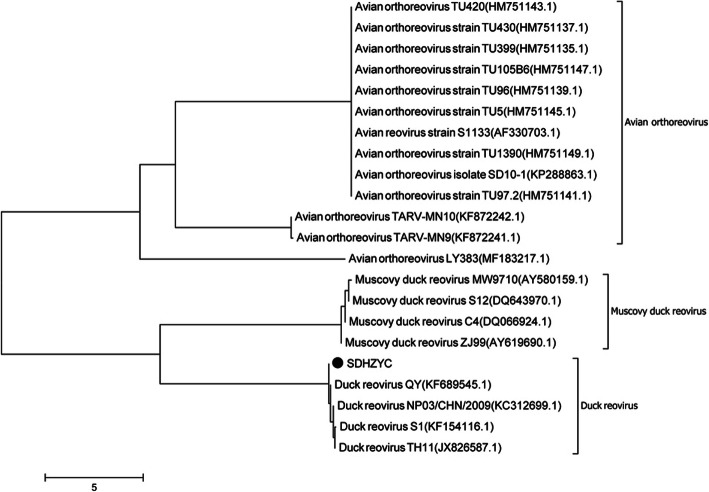
Phylogenetic relationship between SDHZYC(●) and other avian reovirus strains based on the σC gene in the phylogenetic tree. The tree was constructed using the neighbour-joining algorithm of MEGA5.0, and 1000 bootstrap replicates were performed to assign confidences to the groupings. The tree is drawn to scale, with branch lengths in the same units as those of the evolutionary distances used to infer the phylogenetic tree. Note: The SDHZYC strain was marked with filled circles

From GenBank (www.ncbi.nlm.nih.gov), thirteen ARVs, four MDRVs, and four DRVs strains were downloaded to compare the difference among SDHZYC and other DRV, ARV and MDRV strains. The SDHZYC (GenBank accession number MK789277) strain was a new clone from a field isolate. By constructing the phylogenetic tree of σC genes (Fig. 2) and homology analysis, we observed that the SDHZYC strains grouped with China DRV strains. The SDHZYC strain shared 96.9–97.2% sequence similarities with strains QY (KF685545), NP03(KC312699), S1(KF154116) and TH11(JX826587). The SDHZYC strain only shared approximately 41.8% with ARV and 51% with MDRV. These suggest that the genetic evolutionary relationship of the SDHZYC strain is more similar to China DRV strains, and NDRV is caused by a mutation in DRV [18].

### Standard curve, sensitivity and repeatability

**Fig. 3:**
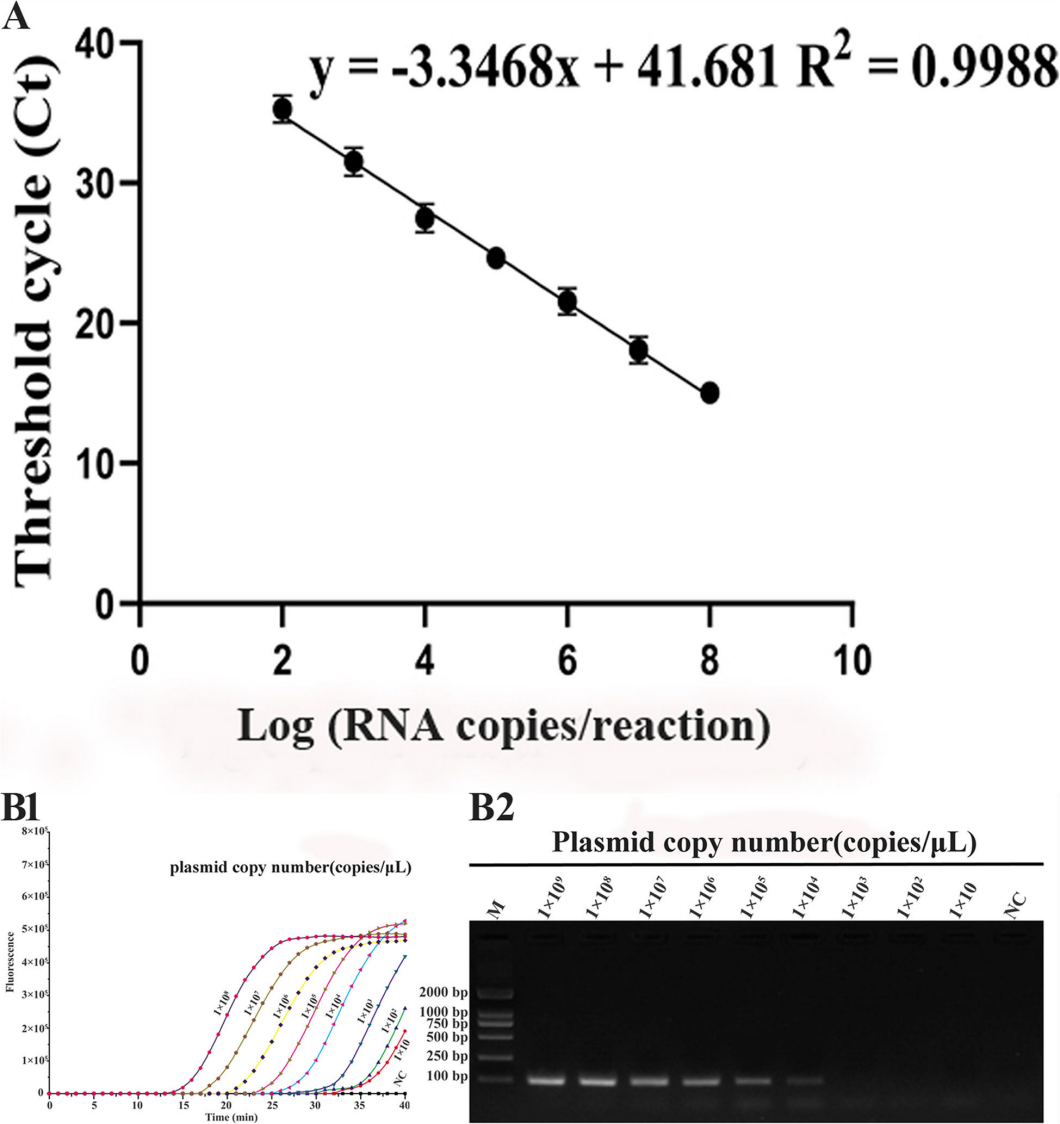
A. Standard curve of the real-time PCR. The triplicate standard curve plots indicate a linear correlation between the Log of the copy number and the CT. The logarithm values (log C) of the detected concentrations of the N-DRV DNA standards (X axis) ranged from 1.0 × 10^8^ to 1.0 × 10^2^ copies/μL, and used the corresponding Threshold cycle (CT value) of each reaction tube fluorescent signal approaching the set threshold (Y axis) of the amplification to perform linear regression. Three replicates were tested for each dilution. B1. Sensitivity of real-time PCR assay for N-DRV detection. B2. Sensitivity of conventional PCR assay for N-DRV detection. M: DL2000 DNA Marker. NC: Nuclease-free water

The results are showed in Fig. 3A, the triplicate standard curve plots indicate a linear correlation between the Log of the copy number and the CT. The standard curve was Y = − 3.3468X + 41.681, of which Y = threshold cycle and X = log sta. The linear correlation (R^2^) of the standard curve was 0.9988. The concentration of plasmid was from 1.0 × 10^8^ to 1.0 × 10^2^ copies/μL. The range of DNA copy numbers of the standard curve was from 1.0 × 10^7.9^ to 1.0 × 10^2.2^ copies/μL.

To evaluate the sensitivity of the RT-qPCR assay, the DNA standards plasmid was diluted from 1.0 × 10^9^ copies/μL to 1.0 × 10^0^ copies/μL. After confirmation, the lowest detection limit of the RT-qPCR was 1.0 × 10^1^ copies/μL (Ct values: 38.3) (Fig. 3B1). By comparison, the lowest detection standard of conventional PCR only was 1.0 × 10^4^ copies/μL (Fig. 3B2).

On 3 different days, 10-fold serial dilutions of standard NDRV plasmid DNA (concentration from 1.0 × 10^8^ to 1.0 × 10^1^ copies/μL) were used to test the intra- and inter-assay reproducibility. All samples were detected in triplicate [19]. In the detection of the intra-assay, the CVs ranged from 0.07 to 1.48%, and the result of the inter-assay CVs ranged from 0.49 to 2.91% (Table 2). It shows that the repeatability of RT-qPCR is high.

**Table 2:** Intra- and inter- assay variability of Ct values of assay in detection of NDRV

Copies of standard plasmid DNA	Intra-assay variability of Ct values				Interassay variability of Ct values			
	Proportion of positive Samples^a^	Ct			Proportion of positive Samples^*^	Ct		
		Mean	SD	CV (%)		Mean	SD	CV (%)
10^8^	1.00	15.04	0.03	0.20	1.00	15.20	0.20	1.32
10^7^	1.00	17.47	0.06	0.34	1.00	17.69	0.17	0.96
10^6^	1.00	20.72	0.23	1.11	1.00	20.50	0.10	0.49
10^5^	1.00	23.94	0.13	0.54	1.00	23.78	0.28	1.18
10^4^	1.00	27.11	0.02	0.07	1.00	27.85	0.78	2.80
10^3^	1.00	30.79	0.45	1.46	1.00	30.91	0.90	2.91
10^2^	1.00	34.32	0.13	0.38	1.00	34.15	0.54	1.58
10	1.00	37.84	0.56	1.48	1.00	37.24	0.35	0.94

^a^Proportion of positive = positive samples/total tested samples (*n* = 3)

### Specificity analysis of the RT-qPCR reaction

**Fig. 4:**
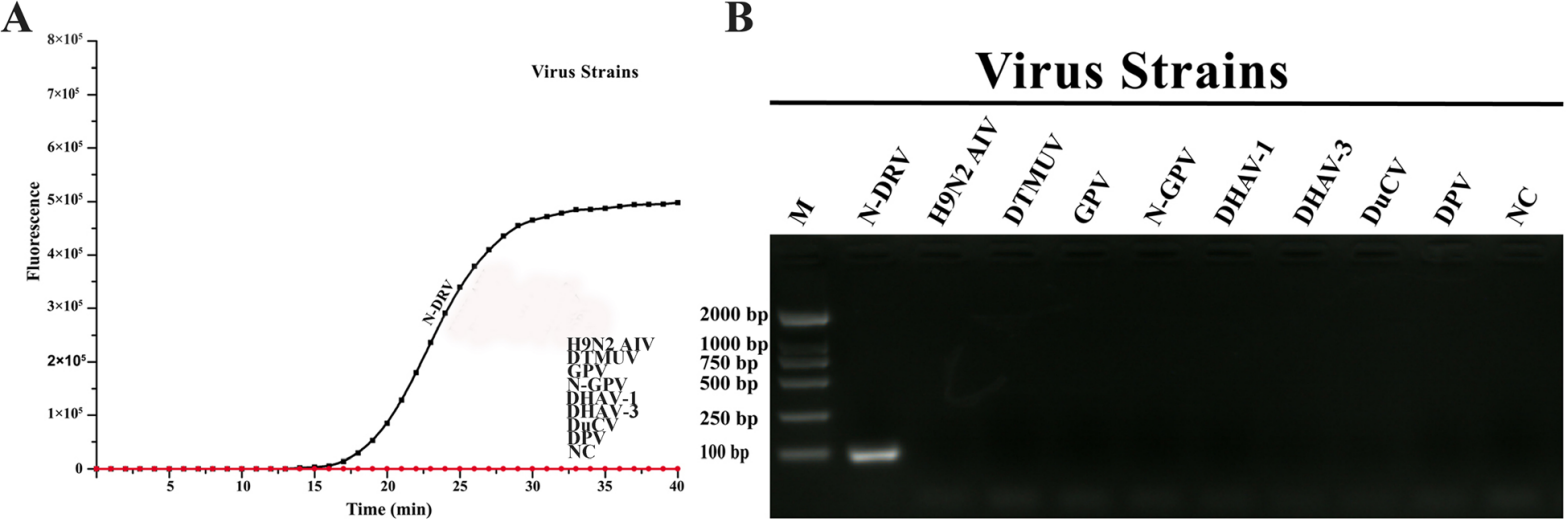
Specificity test results of real-time PCR assay using different virus strains. **a** Amplification plots of different virus strains. **b** Results of Agarose gel electrophoresis. N-DRV: New duck reovirus. H9N2 AIV: Avian influenza virus. DTMUV: Duck tembusu virus. GPV: Goose parvovirus. N-GPV: Novel goose parvovirus. DHAV-1: Duck hepatitis virus type 1. DHAV-3: Duck hepatitis virus type 3. DuCV: Duck circovirus. DPV: Duck Plague Virus. M: DL2000 DNA Marker. NC: Nuclease-free water

Eight different avian viruses were used to test the specificity of RT-qPCR detection. After the detection, NDRV develops a strong response signal. But H9N2 AIV, DTMUV, GPV, N-GPV, DHAV-1, DHAV-3, DuCV, DPV, and Nuclease-free water were not amplified (Fig. 4). The results show that the PCR is specific for NDRV when tested against the listed pathogens.

### Experimentally infected ducklings

**Fig. 5:**
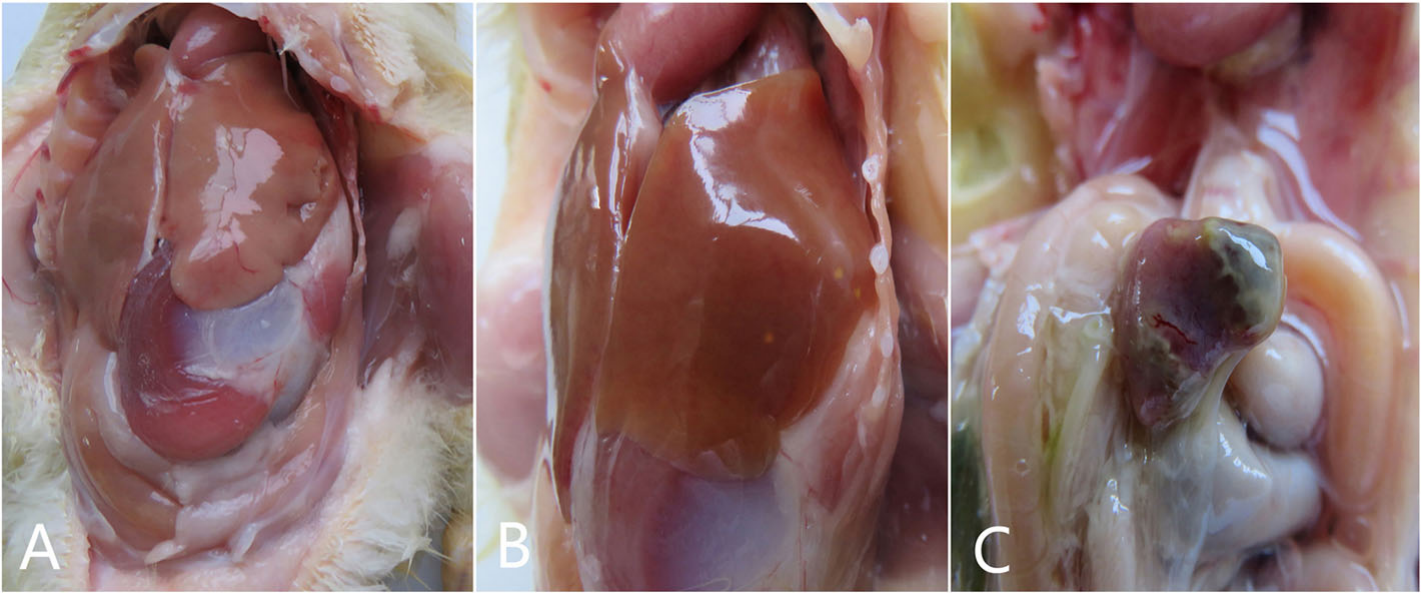
Pathological changes of N-DRV afected 1-day-old ducklings. **a** Control group. **b** Hepatomegaly, bleeding, brittle texture and hemorrhagic necrosis. **c** Splenomegaly, patchy hemorrhagic necrosis

The major pathological changes of the ducklings include enlarged liver hepatomegaly with bleeding and necrosis, brittle texture, red darken, splenomegaly, patchy hemorrhagic necrosis [23] (Fig. 5). Ducks in groups 2 did not show clinical signs. The most important purpose of using experimentally infected ducklings is to validate the RT-qPCR using clinical samples of RT-qPCR. Therefore, we collected thirty-nine samples respectively from different affected organs at 24 h, 48 h and 72 h, including heart, liver, spleen, lung, kidney, pancreas, stomach, brain, intestinal, bursa, thymus, stool and serum. The result was showed in Table 3. After conventional RT-PCR assay and real-time PCR assay, thirty-nine samples of group 2 are negative. Compare to conventional RT-PCR assay, real-time PCR assay detected NDRV earlier, and more frequently. This means that real-time PCR assay had high sensitivity and is appropriate for the detection of NDRV.

**Table 3:** Conventional RT-PCR assay and real-time PCR assay detect thirty-nine samples respectively from different affected organs at 24, 48 and 72 h

		Conventional RT-PCR assay		Real-time PCR assay				
No	Samples	Number of positive/ Number of samples	Positive rate(%)	Number of positive/ Number of samples	Positive rate(%)	viral copy numbers		
1	Heart (24hpi)	0/3	0	0/3	0	Neg.	Neg.	Neg.
2	Liver (24hpi)	0/3	0	0/3	0	Neg.	Neg.	Neg.
3	Spleen (24hpi)	2/3	66.7	2/3	66.7	10^2.8^	Neg.	10^1.7^
4	Lung (24hpi)	3/3	100	3/3	100	10^3.4^	10^4.2^	10^4.9^
5	Kidney(24hpi)	0/3	0	0/3	0	Neg.	Neg.	Neg.
6	Pancreas(24hpi)	0/3	0	0/3	0	Neg.	Neg.	Neg.
7	Stomach(24hpi)	0/3	0	1/3	33.3	Neg.	Neg.	10^0.9^
8	Brain (24hpi)	3/3	100	3/3	100	10^2.7^	10^3.4^	10^2.0^
9	Intestinal(24hpi)	0/3	0	0/3	0	Neg.	Neg.	Neg.
10	Bursa(24hpi)	1/3	33.3	2/3	66.7	Neg.	10^2.9^	10^2.4^
11	Thymus (24hpi)	0/3	0	2/3	66.7	10^3.4^	10^2.5^	Neg.
12	Stool(24hpi)	0/3	0	0/3	0	Neg.	Neg.	Neg.
13	Serum (24hpi)	2/3	66.7	2/3	66.7	10^2.8^	10^3.9^	Neg.
14	Heart (48hpi)	2/3	66.7	3/3	100	10^1.3^	10^2.4^	10^2.6^
15	Liver (48hpi)	1/3	33.3	2/3	66.7	10^2.9^	10^2.1^	Neg.
16	Spleen (48hpi)	3/3	100	3/3	100	10^4.2^	10^3.3^	10^2.5^
17	Lung (48hpi)	3/3	100	3/3	100	10^3.7^	10^2.1^	10^3.0^
18	Kidney(48hpi)	1/3	33.3	2/3	66.7	10^2.6^	10^2.5^	Neg.
19	Pancreas(48hpi)	2/3	66.7	2/3	66.7	10^2.2^	10^2.8^	Neg.
20	Stomach(48hpi)	3/3	100	3/3	100	10^2.3^	10^2.3^	10^2.5^
21	Brain (48hpi)	3/3	100	3 /3	100	10^2.2^	10^3.2^	10^3.1^
22	Intestinal(48hpi)	1/3	33.3	2/3	66.7	10^5.8^	Neg.	10^5.8^
23	Bursa(48hpi)	3/3	100	3/3	100	10^2.3^	10^3.9^	10^2.8^
24	Thymus (48hpi)	1/3	33.3	2/3	66.7	Neg.	10^2.6^	10^3.0^
25	Stool(48hpi)	3/3	100	3/3	100	10^4.7^	10^4.5^	10^4.8^
26	Serum (48hpi)	3/3	100	3/3	100	10^3.5^	10^3.5^	10^3.4^
27	Heart (72hpi)	2/3	66.7	3/3	100	10^2.6^	10^2.8^	10^2.9^
28	Liver (72hpi)	3/3	100	3/3	100	10^2.6^	10^2.5^	10^1.8^
29	Spleen (72hpi)	3/3	100	3/3	100	10^5.4^	10^7.4^	10^5.2^
30	Lung (72hpi)	3/3	100	3/3	100	10^4.9^	10^5.6^	10^4.2^
31	Kidney(72hpi)	3/3	100	3/3	100	10^2.0^	10^3.8^	10^3.2^
32	Pancreas(72hpi)	3/3	100	3/3	100	10^2.4^	10^1.9^	10^3.2^
33	Stomach(72hpi)	3/3	100	3/3	100	10^2.5^	10^3.5^	10^3.1^
34	Brain (72hpi)	3/3	100	3/3	100	10^2.4^	10^4.6^	10^3.1^
35	Intestinal(72hpi)	3/3	100	3/3	100	10^5.4^	10^6.8^	10^6.2^
36	Bursa(72hpi)	3/3	100	3/3	100	10^5.3^	10^6.0^	10^6.3^
37	Thymus (72hpi)	3/3	100	3/3	100	10^2.9^	10^2.9^	10^2.6^
38	Stool(72hpi)	3/3	100	3/3	100	10^6.5^	10^6.2^	10^4.7^
39	Serum (72hpi)	3/3	100	3/3	100	10^3.9^	10^3.9^	10^4.0^

### Clinical sample detection

One hundred and twenty clinical spleen samples from the cherry valley ducklings with NDRV were identified by the TaqMan based real-time PCR and conventional PCR assays. Of these, 113 samples were RT-qPCR positive, and only 101 samples were cPCR positive. The results are presented in Table 4. Statistical analysis showed a difference between the two methods was significant (*P* < 0.05) in the detection of clinical samples. The positive rate of NDRV was 84.17% according to the detection of conventional PCR. However, the positive rate of NDRV was 94.17% through the detection of the RT-qPCR assay established in the study. In the same run described above, samples from non-inoculated SPF chicken embryo were tested negative by RT-qPCR and cPCR. The Copy number of clinical samples were shown in Table 5.

**Table 4:** List of RT-qPCR and conventional RT-PCR results for clinical samples for NDRV

Result by		No. of samples (total, 120)
RT-qPCR	cPCR^a^	
Pos.^b^	Pos.	101
Neg.^c^	Neg.	7
Pos.	Neg.	12
Neg.	Pos.	0

^a^cPCR, conventional RT-PCR

^b^Pos., Positive

^c^Neg., Negative

**Table 5:** Copy number of clinical samples

No	Sample source	CT value	No	Sample source	CT value	No	Sample source	CT value
1	Weifang (Shandong)	16.1241	41	Weifang (Shandong)	13.695	81	Linyi (Shandong)	15.1162
2	Weifang (Shandong)	13.1678	42	Weifang (Shandong)	21.1731	82	Dangshan (Anhui)	13.9539
3	Weifang (Shandong)	25.1597	43	Weifang (Shandong)	24.7179	83	Dangshan (Anhui)	24.9559
4	Weifang (Shandong)	26.5132	44	Weifang (Shandong)	13.0328	84	Dangshan (Anhui)	13.846
5	Weifang (Shandong)	19.0494	45	Weifang (Shandong)	Neg.	85	Dangshan (Anhui)	21.5004
6	Weifang (Shandong)	21.8586	46	Weifang (Shandong)	24.1907	86	Dangshan (Anhui)	15.1966
7	Weifang (Shandong)	25.3857	47	Weifang (Shandong)	20.7851	87	Dangshan (Anhui)	Neg.
8	Weifang (Shandong)	22.4812	48	Weifang (Shandong)	23.6762	88	Dangshan (Anhui)	21.2269
9	Weifang (Shandong)	13.1635	49	Weifang (Shandong)	13.159	89	Dangshan (Anhui)	13.1864
10	Weifang (Shandong)	16.8451	50	Weifang (Shandong)	20.3433	90	Dangshan (Anhui)	18.0723
11	Weifang (Shandong)	21.8283	51	Weifang (Shandong)	Neg.	91	Dangshan (Anhui)	23.813
12	Weifang (Shandong)	22.472	52	Weifang (Shandong)	20.1206	92	Dangshan (Anhui)	Neg.
13	Weifang (Shandong)	20.3765	53	Weifang (Shandong)	20.0127	93	Dangshan (Anhui)	24.0662
14	Weifang (Shandong)	25.9252	54	Weifang (Shandong)	13.0581	94	Dangshan (Anhui)	14.9209
15	Weifang (Shandong)	17.833	55	Weifang (Shandong)	17.0726	95	Dangshan (Anhui)	13.9889
16	Weifang (Shandong)	20.8678	56	Linyi (Shandong)	14.9147	96	Dangshan (Anhui)	24.7853
17	Weifang (Shandong)	23.8947	57	Linyi (Shandong)	21.283	97	Dangshan (Anhui)	21.1174
18	Weifang (Shandong)	16.022	58	Linyi (Shandong)	23.7864	98	Dangshan (Anhui)	19.674
19	Weifang (Shandong)	21.404	59	Linyi (Shandong)	20.1406	99	Dangshan (Anhui)	21.1937
20	Weifang (Shandong)	23.0637	60	Linyi (Shandong)	13.6614	100	Dangshan (Anhui)	17.0916
21	Weifang (Shandong)	14.28	61	Linyi (Shandong)	26.6927	101	Dangshan (Anhui)	15.1441
22	Weifang (Shandong)	14.2534	62	Linyi (Shandong)	17.2705	102	Dangshan (Anhui)	24.7838
23	Weifang (Shandong)	13.925	63	Linyi (Shandong)	19.4452	103	Xuzhou (Jiangsu)	20.488
24	Weifang (Shandong)	13.964	64	Linyi (Shandong)	13.7376	104	Xuzhou (Jiangsu)	17.6883
25	Weifang (Shandong)	24.5377	65	Linyi (Shandong)	26.75	105	Xuzhou (Jiangsu)	24.3292
26	Weifang (Shandong)	24.459	66	Linyi (Shandong)	17.3734	106	Xuzhou (Jiangsu)	15.0757
27	Weifang (Shandong)	24.7221	67	Linyi (Shandong)	13.8337	107	Xuzhou (Jiangsu)	14.9268
28	Weifang (Shandong)	21.422	68	Linyi (Shandong)	17.367	108	Xuzhou (Jiangsu)	13.7254
29	Weifang (Shandong)	21.525	69	Linyi (Shandong)	13.0877	109	Xuzhou (Jiangsu)	17.5209
30	Weifang (Shandong)	21.4153	70	Linyi (Shandong)	23.5349	110	Xuzhou (Jiangsu)	18.5054
31	Weifang (Shandong)	18.3021	71	Linyi (Shandong)	14.8449	111	Xuzhou (Jiangsu)	15.009
32	Weifang (Shandong)	18.3309	72	Linyi (Shandong)	16.8813	112	Xuzhou (Jiangsu)	21.9341
33	Weifang (Shandong)	18.3773	73	Linyi (Shandong)	13.0384	113	Xuzhou (Jiangsu)	16.7821
34	Weifang (Shandong)	15.6995	74	Linyi (Shandong)	24.0116	114	Xuzhou (Jiangsu)	18.1889
35	Weifang (Shandong)	Neg.	75	Linyi (Shandong)	14.8421	115	Xuzhou (Jiangsu)	17.4116
36	Weifang (Shandong)	15.7435	76	Linyi (Shandong)	17.9888	116	Xuzhou (Jiangsu)	Neg.
37	Weifang (Shandong)	15.7018	77	Linyi (Shandong)	19.8084	117	Xuzhou (Jiangsu)	17.7065
38	Weifang (Shandong)	13.6976	78	Linyi (Shandong)	15.133	118	Xuzhou (Jiangsu)	13.7366

## Discussion

In China, NDRV has emerged in recent years and is a current common genotype [13, 16]. Recently, a group of newly emerging DRVs [24–26] was confirmed and characterized in Cherry Valley duck in China [18]. The NDRV from the mainly infected ducklings caused hemorrhage and necrosis in the liver. There are several notable different properties between classical MDRV and NDRV, including different antigenicity by cross-neutralization tests, host species differences, pathogenic properties, protein profiles [27–29], electropherotypes, and genomic coding assignments [10–13, 16, 30]. As fatal pathogenic viruses that can kill ducklings within 72 h, NDRV had caused huge economic losses for the duck industry over the past several decades [31]. Therefore, an easy rapid highly sensitive and specific method for NDRV detection is crucially required to develop [23].

In this study, we designed the probe and primers used in the study based on the S2 gene of NDRV. By using the mVISTA online program, we found that primers (NDRV-F and NDRV-R) and probe (NDRV-P) distinguished duck reovirus from other reoviruses. Then, a TaqMan-based real-time PCR for detecting NDRV infection was established. Verified by a series of experiments, the RT-qPCR has high sensitivity, specificity, and reproducibility. The sensitivity of the RT-qPCR was evaluated using ten-fold diluted DNA standard plasmid, and the lowest amount of detection for NDRV was found was 1.0 × 10^1^ copies/μL (Ct values: 38.3). It’s thousands of times higher than conventional PCR (1.0 × 10^4^ copies/μL). In subsequent experiments, the RT-qPCR showed high analytical specificity because other duck-derived pathogens were not detected, including Avian influenza virus (H9N2 AIV), Duck Tembusu virus (DTMUV), Goose parvovirus (GPV), Novel goose parvovirus (N-GPV), Duck hepatitis virus type 1 and 3 (DHAV-1 and DHAV-3), Duck circovirus (DuCV), Duck Plague Virus (DPV). The RT-qPCR assay was also found to be highly reproducible. The variability of intra-assay and inter-assay were ≤ 1.48 and 2.91%, respectively.

The performance of the RT-qPCR assay used as a diagnostic tool to rapidly detect the NDRV is confirmed by the tested results using one hundred and twenty clinical specimens from suspected cases of infected ducks from different regions of China. These clinical samples, spleen samples, were obtained from different duck farms and laboratory diagnostic cases. Comparative analysis of the conventional PCR and RT-qPCR assay using clinical samples showed significant differences. The positive rate of infection of conventional PCR was merely 84.17% while RT-qPCR was 94.17%. This has demonstrated the higher sensitivity of the TaqMan-based real-time PCR.

## Conclusions

The RT-qPCR could be used as a reliable tool for the rapid detection of NDRV clinical samples, thereby facilitating epidemiological investigations of animals infected with NDRV.

## Methods

### Virus isolation

In this study, we isolated the reovirus from Cherry Valley duck [18] in Shandong province, China. The DRV field strain were isolated from spleen tissues of sick bird which showed symptoms of NDRV infections. The necrotic spleen tissue was extracted from sick birds, homogenized in phosphate-buffered saline (PBS, pH 7.2), freeze-thawed three times, and centrifuged at 8000×g for 15 min [18, 32]. The virus was isolated in LMH (Leghorn Male-chicken Hepatocellular-carcinoma, ATCC CRL-2013) cell and named SDHZYC. The cultures were incubated at 37 °C with 5% CO_2_ and checked daily for giant or bloom-like cytopathic effects (CPEs). The virus was collected when we observed more than 80% CPEs. Then, we subculture virus until a stable CPE could be harvested and stored at − 80 °C [18, 32]. The institute of avian disease in Shandong Agricultural University propagated other avian viruses (Avian influenza virus (H9N2 AIV), Duck Tembusu virus, Goose parvovirus, Novel goose parvovirus, Duck hepatitis virus type 1 and 3, Duck circovirus, Duck Plague Virus) in of 9- to 11-day-old embryonated specific-pathogen-free (SPF) eggs (Poultry research institute, Shandong Academy of Agricultural Sciences, Jinan, China) through chorioallantoic membrane route or chorioallantoic sac route [32].

### Experimental infection of ducklings

Eighteen 1-day-old cherry valley ducklings have divided ducklings into 2 groups (9 ducklings in each group) randomly. To study the NDRV infection, group 1 was intramuscularly injected with 0.2 mL (10^6.367^ ELD_50_) of the NDRV cell fluid. As the control group, group 2 was treated with sterile DMEM (500 mL, Catalog 01–172-1ACS; BI, Shanghai, China) in the same way. All ducklings were purchased from the commercial hatchery of Yike Company Limited in Xintai County. All experiments with ducks were fed and managed at Shandong Agricultural University according to the established humane procedures and biosecurity guidelines. Water and food were fed ad libitum and were provided living conditions of 40–60% relative humidity and a 12/12 h light/dark cycle every day. All ducklings were observed and euthanatized using intravenous pentobarbital sodium (New Asia Pharmaceutical, Hainan, China) for 72 h post-infection (hpi) [23].

### RNA and DNA extraction

Total RNAs (RNA viruses, i.e. H9N2 AIV, DTMUV, DHAV-1 and DHAV-3, NDRV) were extracted by MiniBEST Universal RNA Extraction Kit (50 preps, Catalog DP430; TIANGEN, Beijing, China) following the manufacturer’s instructions. Total DNAs (DNA viruses, i.e. GPV, N-GPV, DuCV, and DPV) were extracted using TIANamp Genomic DNA Kit (50 preps, Catalog DP304–02; TIANGEN, Beijing, China) according to instructions provided by the manufacturer [33]. All extracted RNAs and DNAs templates were stored at − 80 °C until use.

### Sequence analysis

According to reports [31], the sigma C is the major antigenic determinant of avian reovirus. Structural protein Sigma C was the main protein of avian reovirus. It is in the shell of the virus, carried the surface antigen of virus type-specific neutralization reaction. It is related to the adsorption, proliferation, and syncytial formation of the virus. Therefore, it is of great significance to analyze the genetic evolution of Sigma C protein. The S1 segment encoding sigma C gene of NDRV was amplified by primers σC full-length (Forward primer) and σC full-length (Reverse primer) (Table 1). Amplified PCR products were separated on a 1% agarose gel and then purified using the Agarose Gel DNA Purification Kit (200 preps, Catalog D2500–02; OMEGA, Georgia, USA). The PCR products were cloned into the pMD18-T vector (20 preps, Catalog 6011; Takara, Beijing, China) and transformed the positive recombinant plasmid into DH5α competent cells (10 × 100 μL, Catalog BC102–01; Biomed, Beijing, China). Then, the samples were sent to the Beijing Genomics Institute to be sequenced. The sequencing sequences were assembled into a complete 966 bp sequence using the SeqMan program of the DNAstar software package (version 7.1) (DNAstar, Madison, WI, USA). Afterward, the sequence was aligned with other reovirus sequences using the MegAlign program of the DNA star package [34]. Utilizing the neighbor-joining method, a phylogenetic tree was constructed with MEGA 6.0 and performed 1000 bootstrap replicates. In Table 6, all the reference avian reovirus isolates were listed.

**Table 6:** Description of the Avian reovirus isolates involved in this study

Isolates	Accession number	Host	Country
TU399	HM751135	Avian	Tunis
TU430	HM751137	Avian	Tunis
TU96	HM751139	Avian	Tunis
TU97.2	HM751141	Avian	Tunis
TU420	HM751143	Avian	Tunis
TU5	HM751145	Avian	Tunis
TU105B6	HM751147	Avian	Tunis
TU1390	HM751149	Avian	Tunis
S1133	AF330703	Avian	Spain
SD10–1	KP288863	Avian	China
TARV-MN9	KF872241	Avian	USA
TARV-MN10	KF872242	Avian	USA
LY383	MF183217	Avian	China
MW9710	AY580159	Muscovy duck	China
ZJ99	AY619690	Muscovy duck	China
C4	DQ066924	Muscovy duck	China
S12	DQ643970	Muscovy duck	China
TH11	JX826587	Duck	China
NP03	KC312699	Duck	China
QY	KF689545	Duck	China
S1	KF154116	Duck	China
SDHZYC	MK789277	Duck	China

### RT-qPCR assay for NDRV

Based on the obtained fluorescence and lowest threshold cycle (Ct), the concentrations of the primers, probe, and templates were optimized [35]. The optimized RT-qPCR of NDRV was reacted in a 20 μL system (One Step PrimeScript™ RT-PCR Kit; Takara, Beijing, China). It contained 10 μL 2 × One-Step RT-PCR Buffer III, 0.4 μL TaKaRa Ex Taq HS (5 U/μL), 0.4 μL PrimeScript RT Enzyme Mix II (200 units/μL), 0.4 μL Realtime PCR forward primer (10 μM), 0.4 μL Realtime PCR reserve primer (10 μM), 0.8 μL Realtime PCR probe (10 μM), 0.4 μL ROX Reference Dye (50×), 5.6 μL ddH_2_O, and 2.0 μL RNA template. The RT-qPCR was conducted with Applied Biosystems® 7300 FAST Real-Time PCR System. The reaction conditions include 42 °C for 5 min and 95 °C for 10 s, 40 cycles at 95 °C for 5 s, and 60 °C for 20 s. During the extension step, Fluorescent signals were collected. We analyzed the result of each assay with Sequence Detector software (version 2.1; Applied Biosystems).

### Standard plasmid preparation, construct standard curves and sensitivity

The forward primer (RT-qPCR-F) and reverse primer (RT-qPCR-R) were used to amplify the partial S2 gene (85 bp) of NDRV. The PCR products were separated by electrophoresis on 1.0% agarose gel. The PCR product was cloned into pMD18-T (the plasmid vector) and then was verified by sequencing [36]. The plasmid of pMD18-NDRV was serially diluted from 1.0 × 10^10^ copies/μL to 1.0 × 10^1^ copies/μL by 10 × Tris-EDTA Buffer (pH 7.4), and stored at − 20 °C. The 10 × Tris-EDTA Buffer (pH 7.4) is prepared by Tris-EDTA Buffer 10 × Powder, pH 7.4 (10 pouches, Catalog T9111; Takara, Beijing, China) dissolved in water. Then, it was used to construct the standard curve [19] and confirm the detection limit of RT-qPCR.

### Conventional PCR for NDRV

Meanwhile, conventional PCR (cPCR) was conducted [37] under the same circumstances. The primers used for the cPCR were showed in Table 1. The reaction conditions include 95 °C for 5 min, 35 cycles at 95 °C for 30 s, 55 °C for 30 s and 72 °C for 30 s; and 72 °C for 5 min at last. The sensitivity of the cPCR was confirmed by agarose gel electrophoresis. Nuclease-free water was used as the negative control in RT-qPCR determination. And all reactions were repeated three times.

### Specificity analysis of the RT-qPCR reaction

Other duck-derived viruses were used to prove the specificity of the RT-qPCR reaction, including Avian influenza virus (H9N2 AIV), Duck tembusu virus (DTMUV), Goose parvovirus (GPV), Novel goose parvovirus (N-GPV), Duck hepatitis virus type 1 and 3 (DHAV-1 and DHAV-3), Duck circovirus (DuCV), Duck Plague Virus (DPV). The RT-qPCR assay was performed in triplicate.

### Repeatability analysis of the RT-qPCR assay

To evaluate the coefficient of variation (CV) of the RT-qPCR, the 10-fold dilutions of pMD18-S2 (concentration of 1.0 × 10^8^ to 1.0 × 10^1^ copies/μL) were tested. In checking to see the intra-batch repeatability, triplicates of each dilution were detected, and according to the formula of the geometric mean Cq values / standard deviation calculated the coefficients of variation (CV). The coefficient of variation for inter-assay repeatability shows the differences among the measures at different times [33]. Three repeats were performed for each of the inter and intra assay analysis.

### Clinical samples detection

During 2017–2018, we collected 120 samples of spleen from suspected cases of infected ducks. According to the survey, these samples were collected from different regions of Weifang (Shandong), Linyi (Shandong), Dangshan (Anhui), and Xuzhou (Jiangsu). One hundred and twenty clinical spleen samples were detected with the TaqMan based real-time PCR and conventional PCR assays (Primers and probe were presented in Table 1). The tissue samples from SPF duck embryo were used as controls.

### Statistical analysis

Statistically significant differences in mean detection rates were determined by one-way ANOVA assessment using GraphPad Prism version 6 (GraphPad Software Inc., San Diego, Calif.) when different types of samples were tested. At *P < 0.05, the difference was considered significant.

## Data Availability

All data generated or analyzed during this study are included in this article. The datasets (RNA sequencing data, GenBank accession number MK335954) generated and/or analysed during the current study are available in the NCBI.

## Abbreviations

ARV: Avian reovirus
cPCR: Conventional PCR
CPEs: Cytopathic effects
Ct: Threshold cycle
CV: Coefficient of variation
DHAV-1: Duck hepatitis virus Ι
DHAV-3: Duck hepatitis virus ΙΙΙ
DPV: Duck plague virus
DRV: Duck reovirus
DTMUV: Duck tembusu virus
DuCV: Duck circovirus
GPV: Goose parvovirus
H9N2 AIV: H9 subtype avian influenza
LMH: Leghorn Male-chicken Hepatocellular-carcinoma
MDRV: Muscovy Duck Reovirus
NDRV: New duck reovirus
N-GPV: New goose parvovirus
RT-qPCR: TaqMan-based real-time PCR assay
